# Immune-Associated Proteins Are Enriched in Lung Tissue-Derived Extracellular Vesicles during Allergen-Induced Eosinophilic Airway Inflammation

**DOI:** 10.3390/ijms22094718

**Published:** 2021-04-29

**Authors:** Cecilia Lässer, Yasunari Kishino, Kyong-su Park, Ganesh Vilas Shelke, Nasibeh Karimi, Shintaro Suzuki, Lilit Hovhannisyan, Madeleine Rådinger, Jan Lötvall

**Affiliations:** 1 Krefting Research Centre, Institute of Medicine, at Sahlgrenska Academy, University of Gothenburg, 405 30 Gothenburg, Sweden; ookiyookiy@med.showa-u.ac.jp (Y.K.); kyong-su.park@gu.se (K.-s.P.); ganesh.shelke@gu.se (G.V.S.); nasibeh.karimi@gu.se (N.K.); szshintr@med.showa-u.ac.jp (S.S.); lilithov@gmail.com (L.H.); madeleine.radinger@lungall.gu.se (M.R.); jan.lotvall@gu.se (J.L.); 2 Section of Respiratory Medicine and Allergology, Department of Internal Medicine, School of Medicine, Showa University, Tokyo 142-8666, Japan; 3 Sahlgrenska Cancer Center, Departments of Surgery, Oncology or Transplantation Surgery, Institute of Clinical Sciences at University of Gothenburg and Sahlgrenska University Hospital, 405 30 Gothenburg, Sweden; 4 Neurosciences and Cellular and Structural Biology Division, Eunice Kennedy Shriver National Institute of Child Health and Human Development, National Institutes of Health, Bethesda, MD 20892-3758, USA; 5 Laboratory of Human Genomics and Immunomics, Institute of Molecular Biology, National Academy of Sciences of Armenia, Yerevan 0014, Armenia

**Keywords:** asthma, allergy, exosomes, extracellular vesicles, quantitative proteomics, tandem mass tag, tissue-derived extracellular vesicles

## Abstract

Studying the proteomes of tissue-derived extracellular vesicles (EVs) can lead to the identification of biomarkers of disease and can provide a better understanding of cell-to-cell communication in both healthy and diseased tissue. The aim of this study was to apply our previously established tissue-derived EV isolation protocol to mouse lungs in order to determine the changes in the proteomes of lung tissue-derived EVs during allergen-induced eosinophilic airway inflammation. A mouse model for allergic airway inflammation was used by sensitizing the mice intraperitoneal with ovalbumin (OVA), and one week after the final sensitization, the mice were challenged intranasal with OVA or PBS. The animals were sacrificed 24 h after the final challenge, and their lungs were removed and sliced into smaller pieces that were incubated in culture media with DNase I and Collagenase D for 30 min at 37 °C. Vesicles were isolated from the medium by ultracentrifugation and bottom-loaded iodixanol density cushions, and the proteomes were determined using quantitative mass spectrometry. More EVs were present in the lungs of the OVA-challenged mice compared to the PBS-challenged control mice. In total, 4510 proteins were quantified in all samples. Among them, over 1000 proteins were significantly altered (fold change >2), with 614 proteins being increased and 425 proteins being decreased in the EVs from OVA-challenged mice compared to EVs from PBS-challenged animals. The associated cellular components and biological processes were analyzed for the altered EV proteins, and the proteins enriched during allergen-induced airway inflammation were mainly associated with gene ontology (GO) terms related to immune responses. In conclusion, EVs can be isolated from mouse lung tissue, and the EVs’ proteomes undergo changes in response to allergen-induced airway inflammation. This suggests that the composition of lung-derived EVs is altered in diseases associated with inflammation of the lung, which may have implications in type-2 driven eosinophilic asthma pathogenesis.

## 1. Introduction

Extracellular vesicles (EVs) are nano-sized membrane-enclosed structures that are released by cells into their surroundings [1], and they contain functional RNAs, proteins, and lipids that can be shuttled from one cell to another, thereby influencing the activity of the recipient cells. EVs have been shown to be involved in cell-to-cell communication both in physiological and pathological states [1,2,3]. For example, immune system EVs secreted from B cells can induce T cell proliferation, dendritic cell-derived EVs can activate natural killer (NK) cells and stimulate cytokine production in T cells, and EVs from mast cells can activate dendritic cells and B and T cells [2].

Asthma is a common respiratory disease in both children and adults and affects about 350 million people worldwide [4]. Asthma is a chronic inflammatory disease of the airway that is characterized by symptoms such as reversible airflow obstruction and episodes of coughing, wheezing, chest tightness, and shortness of breath. Although the role of EVs in different contexts of inflammation has been studied previously, less is known about the role of EVs in allergic inflammation and asthma. It has previously been shown that microRNAs in bronchoalveolar lavage fluid (BALF)-derived EVs are altered in asthma patients [5], that BALF-derived EVs from asthma patients can contribute to bronchial epithelial cell-derived cytokine and leukotriene production [6], and that eosinophil-derived EVs contribute to tissue remodeling in asthma patients by activating structural lung cells [7]. Furthermore, mesenchymal stem cell-derived EVs have been suggested to attenuate allergic asthma in a mouse model [8]. In addition, we recently showed that more EVs are released when air-liquid interphase-cultured human bronchial epithelial cells are stimulated with cytokines as seen in disease-relevant type 2 (T2) and type 17 (Th17) immune responses [9]. Notably, T2 (IL-4 and IL-13), and Th17 (IL-17A and TNF-α) cytokine-stimulated bronchial epithelial cells released EVs with significantly altered proteomes compared to non-activated cells. Additionally, we have recently published several studies using a protocol to isolate EVs directly from metastatic melanoma tissue samples [10,11,12].

Our hypothesis is that the phenotypes of lung EVs undergo significant changes during allergic airway inflammation. To test this, we used our previously published tissue-derived EV isolation protocol [12]. We harvested EVs from allergen-sensitized mice that were challenged with allergen or vehicle, and we performed quantitative proteome analysis to determine how the proteomes were altered in vivo during allergic asthma-like inflammation.

## 2. Results

### 2.1. EVs Can Be Isolated Directly from Lung Tissue

**Figure 1:**
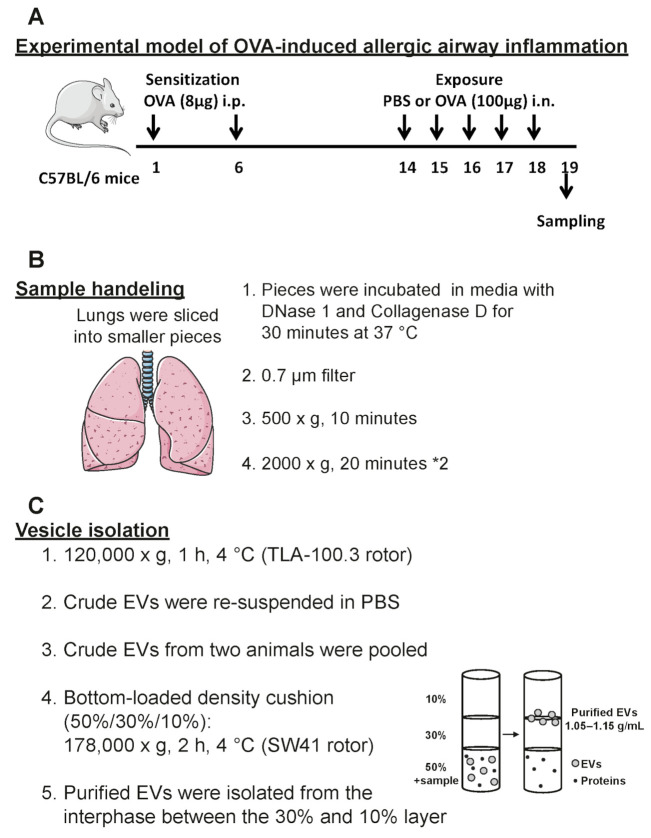
Schematic overview of the experimental workflow. (**A**) Experimental model of OVA-induced allergic airway inflammation. (**B**) Handling and processing of the lung tissue according to our previous protocol [12]. (**C**) Protocol for the isolation of EVs from the lung tissue.

**Figure 2:**
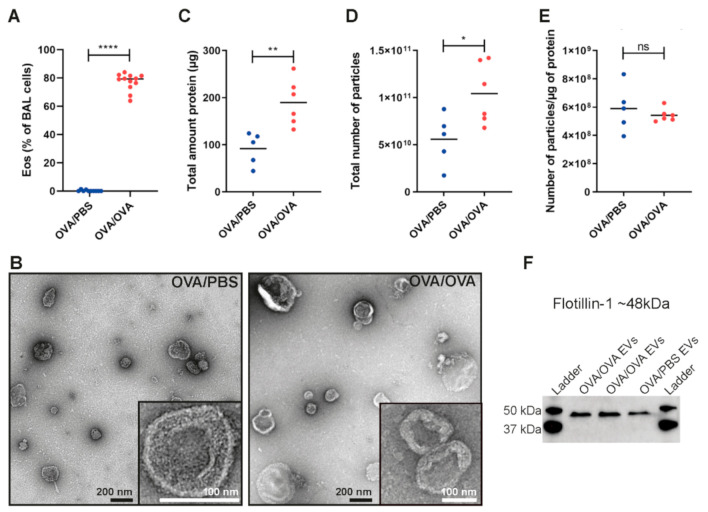
EVs can be isolated from lung tissue. (**A**) Percentage of eosinophils in BALF. *N* = 10–12. (**B**) PBS-EVs and OVA-EVs evaluated with transmission electron microscopy. Scale bars are 200 nm in the full pictures and 100 nm in the magnifications. (**C**,**D**) The total amount of EV protein (**C**) and the number of particles (**D**) was determined in all samples with Qubit and ZetaView, respectively. (**E**) Particle-to-protein ratio for all samples. *N* = 5–6 for panels (**C**–**E**). Significance was tested with Student’s non-paired *t*-test. * *p* < 0.05, ** *p* < 0.01, **** *p* < 0.0001, ns; non-significant. (**F**) Two microgram of proteins were loaded per sample and the presence of flotillin-1 was determined with Western blot.

To determine how the proteomes of EVs in the airway are altered upon allergic airway inflammation, such as asthma, we used an experimental mouse model of allergen-induced inflammation (Figure 1A). We have used this model for several years in the group and have previously shown that it induces eotaxin production, eosinophil infiltration, and the production of type 2 cytokines such as IL-13 in the lung [13,14,15]. Differential cell count showed elevated levels of BALF eosinophils exclusively in ovalbumin (OVA)-sensitized and OVA-challenged mice (OVA/OVA) compared to control mice sensitized with OVA and challenged with PBS (OVA/PBS), thus validating that our model presented with an appropriate inflammatory response (Figure 2A). EVs were isolated directly from the lung tissue from both OVA/PBS and OVA/OVA mice in a similar manner as previously published by us (10–12) with minor changes (Figure 1B,C). Electron microscopy showed that EVs with the diameters of 50–400 nm were isolated from the lungs of both OVA/PBS and OVA/OVA mice (Figure 2B). Importantly, more EVs could be isolated from the OVA/OVA lung tissue compared to the OVA/PBS lung tissue as measured by EV protein levels and EV particles numbers (Figure 2C,D). The particle-to-protein ratio has been suggested as a measurement of the purity of isolated EVs [16], and no difference in purity was observed between the OVA/PBS and OVA/OVA EVs (Figure 2E). Western blot showed that EVs from both OVA/PBS and OVA/OVA mice contained flotillin-1 (Figure 2F).

**Figure 3:**
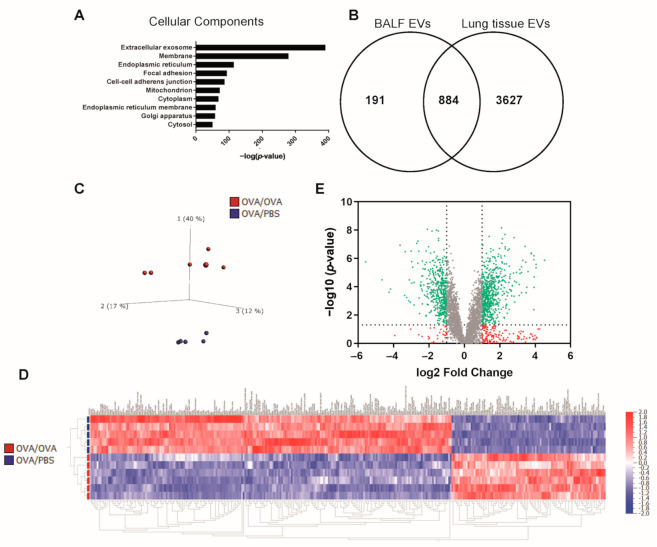
The proteome of lung tissue-derived EVs is altered upon allergen stimulation. Quantitative proteomics (TMT) was used to determine the differences in lung tissue-derived EVs under allergen-induced inflammation compared to controls. *N* = 5–6. (**A**) DAVID was used to determine the most-enriched cellular components associated with all proteins quantified in both PBS-EVs and OVA-EVs. (**B**) Our lung tissue-derived EV proteome was compared to a previously published proteome from BALF-derived EVs [17]. (**C**) Principle component analysis illustrating the relationship between PBS-EVs (blue) and OVA-EVs (red). (**D**) A multi-group comparison was performed in Qlucore and showed that 321 proteins were differentially expressed in our dataset (*p* = 0.0001). The list of these proteins is shown in Supplementary Table S2. (**E**) Volcano plot comparing the PBS-EVs and OVA-EVs. The dotted lines indicate cut offs, which are 1.3 on the Y-axis (corresponding to *p* < 0.05) and 1.0 on the X-axis (corresponding to fold change >2).

The protein content of lung tissue-derived EVs was analyzed with quantitative proteomics. A total of 4510 proteins were quantified in all samples (Supplementary Table S1). The identified proteins were analyzed with DAVID to identify enriched cellular components compared to their frequency in the genome, and the top associated terms were “Extracellular exosome” and “Membrane” (Figure 3A), with 33% (1478 proteins) and 58% (2592 proteins) of the identified proteins being associated with the terms, respectively. Several proteins that have previously been found to be present in EVs were among the proteins quantified in the lung-derived EVs (Table 1). This supports the conclusion that we had, indeed, isolated EVs. Next, we compared the proteome of our lung tissue-derived EVs isolated here with a previously published proteome of mouse BALF-derived EVs by another research group [17]. Our lung tissue-derived EVs contained 82% of the proteins previously identified in these BALF-derived EVs (Figure 3B), and we quantified an additional 3627 proteins not previously identified in the BALF-derived EVs.

Together, these results show that EVs can be isolated directly from mouse lung tissue and that the lung-tissue EV proteome corresponds well to that of previously published EV studies.

### 2.2. Mitochondrial Proteins Are Decreased in Lung EVs upon Allergen-Induced Inflammation

A principle component analysis was performed in order to visualize the relationship between EVs isolated during inflammation (OVA/OVA condition, hereafter called OVA-EVs) and under control conditions (OVA/PBS condition, hereafter called PBS-EVs) in the lung. A plot of the first three components revealed that component 1, representing 40% of the variability, distinguished the OVA-EVs from the PBS-EVs (Figure 3C). Furthermore, multi-group comparison showed that 321 proteins were significantly (*p* = 0.0001) upregulated or downregulated in the OVA-EVs compared to the PBS-EVs (Figure 3D and Supplementary Table S2). This clear distinction between the inflammation-derived EVs and the control lung tissue-derived EVs was further demonstrated in a volcano plot (Figure 3E). Over 1000 proteins were significantly altered (fold change >2 and *p* < 0.05), with 614 proteins being increased and 425 proteins being decreased in OVA-EVs compared to PBS-EVs.

**Figure 4:**
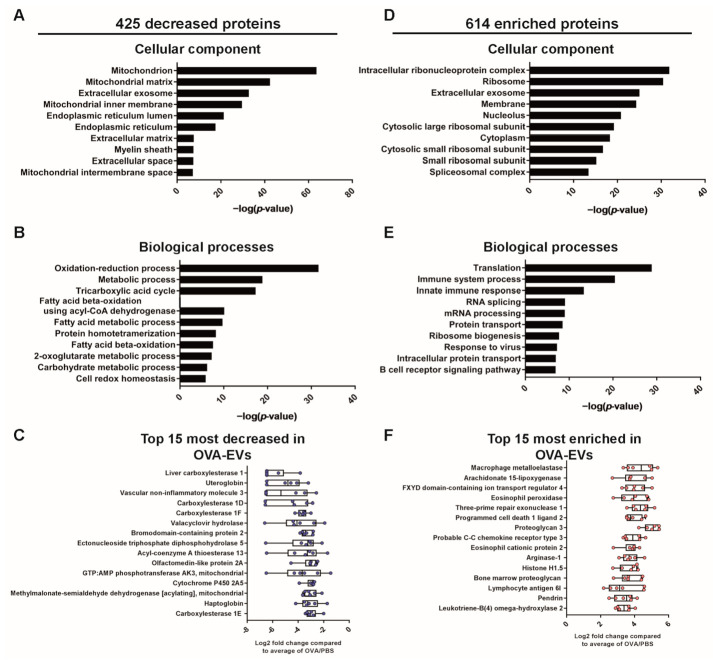
Decreased proteins in OVA-EVs are associated with mitochondria. GO terms associated with the 425 decreased and 614 enriched proteins from the volcano plot in Figure 3E. (**A**,**B**) DAVID was used to determine the most enriched cellular components (**A**) and biological processes (**B**) among the 425 decreased proteins in OVA-EVs. (**C**) The 15 most decreased proteins in OVA-EVs. (**D**,**E**) DAVID was used to determine the most enriched cellular components (**D**) and biological processes (**E**) among the 614 enriched proteins in OVA-EVs. (**F**) The 15 most enriched proteins in OVA-EVs.

The 425 proteins that were decreased during allergen-induced airway inflammation were mainly associated with the mitochondria, endoplasmic reticulum, and the extracellular matrix (Figure 4A). Furthermore, the downregulated proteins were associated with the biological functions “Oxidation-reduction process”, “Metabolic process”, and “Tricarboxylic acid cycle” (Figure 4B), suggesting that these processes are reduced in the EV-producing cell and/or that these proteins are less likely to be loaded into lung EVs during allergen-induced airway inflammation. The majority of the 15 most-decreased proteins were associated with these metabolic and oxidation functions (Figure 4C). Interestingly, the protein uteroglobin, which is primarily produced by the non-ciliated bronchial epithelial cells called club cells, were among the most strongly decreased proteins in the OVA-EVs. The role of uteroglobin is not completely understood, but this could indicate that club cells are a cell type in the lung that releases fewer EVs during inflammation.

### 2.3. The Lung EV Proteome Is Associated with Immune-Related Functions upon Allergen-Induced Airway Inflammation

**Figure 5:**
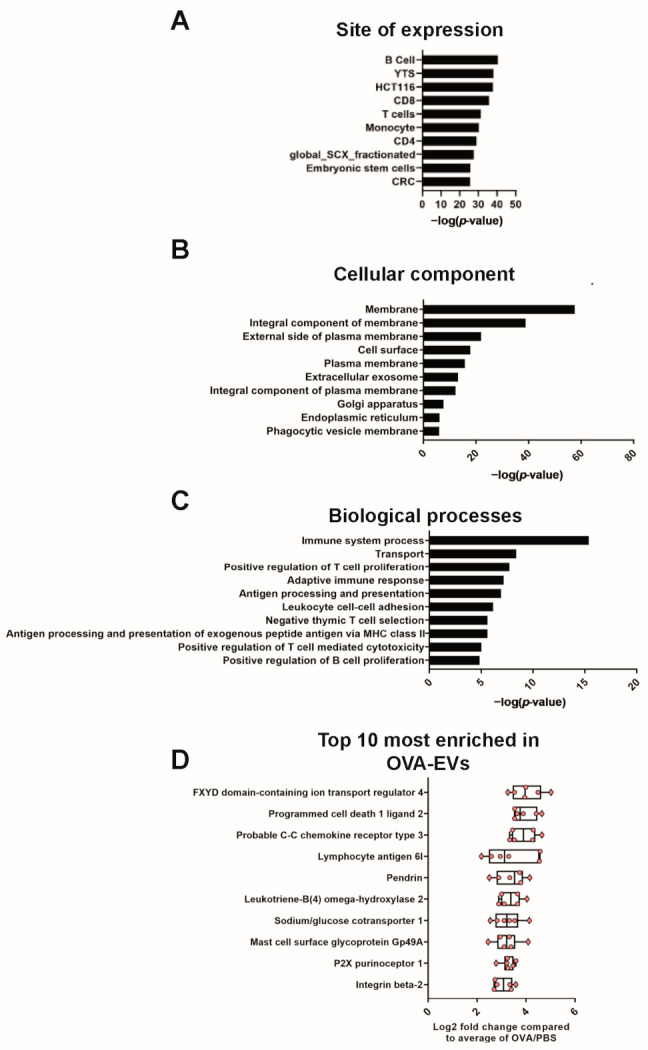
Enriched proteins in OVA-EVs are associated with immune cells and inflammation. (**A**) The 614 enriched proteins in OVA-EVs were analyzed with FunRich to determine the site of expression of these proteins. YTS, natural killer cell line; HCT116, colorectal carcinoma cell line; CRC, colorectal cancer cells. (**B**,**C**) DAVID was used to determine the most enriched cellular components (**B**) and biological processes (**C**) among the 145 enriched membrane proteins in OVA-EVs. (**D**) The 15 most enriched membrane proteins in OVA-EVs.

The 614 enriched proteins in lung tissue-derived EVs during allergen-induced airway inflammation (Figure 3D) were mainly associated with the ribosomal subunits, translation, and mRNA processing, which suggests increased production of proteins in the EV-producing airway cells and/or increased loading of these proteins into EVs (Figure 4D,E). Furthermore, the enriched proteins were also associated with “Immune system processes”, “Innate immune response”, “Response to virus”, and “B cell receptor signaling pathway” (Figure 4E). The majority of the 15 most-enriched proteins were associated with the immune-related terms (Figure 4F). Examples of such proteins are macrophage metalloelastase (also called MMP-12), pendrin, arachidonate 15-lipoxygenase (also called ALOX15), C-C chemokine receptor type 3 (also called CCR3), eosinophil peroxidase and eosinophil cationic protein 2. To determine which cells released the EVs into the inflamed lung, we used bioDBnet to convert the 614 enriched EV proteins from mouse to human accession-IDs. This enabled analysis with FunRich and allowed the determination of the site of expression of these proteins. The proteins enriched in OVA-EVs were mainly expressed in immune cells such as B cells, T cells, monocytes, and NK cells (Figure 5A). 

Next, we downloaded the associated cellular component from the UniProt database for all of the proteins in our dataset. The proteins that either had a transmembrane part or were associated with the membrane through a lipid anchor were analyzed in more depth. This was performed to only include true membrane proteins because proteins can be listed as membrane associated without being a true membrane-bound protein. Out of the 614 enriched proteins in the OVA-EVs, 145 were classified as membrane proteins using this definition and, as expected, when the cellular components of these 145 proteins were analyzed, the top association was with membranes (Figure 5B). Interestingly, the top GO terms were associated with “External side of plasma membrane”, “Cell surface”, “Plasma membrane”, and “Integral component of plasma membrane”, suggesting that the OVA-EVs primarily originated from the plasma membrane and not from organelles such as the nucleus, endoplasmic reticulum, or Golgi apparatus. The biological processes associated with the 145 enriched membrane proteins were largely immune-related, such as “Immune system process”, “Positive regulation of T cell proliferation”, “Adaptive immune responses”, “Antigen processing and presentation”, and “Leukocyte cell–cell adhesion” (Figure 5C). The immune association of the membrane proteins was also clear when the 10 most-enriched membrane proteins in the OVA-EVs were analyzed (Figure 5D).

Taken together, these results argue that EVs are increased in the lung during allergen-induced inflammation and that these EVs are most likely released by the infiltrating immune cells.

## 3. Discussion

We and others have previously shown that EVs can be isolated from different types of tissues, but these studies have mainly focused on determining the cargo and role of EVs from brain tissue [18,19,20,21] and tumor tissue [10,11]. The current study builds on these findings by showing for the first time that EVs can also be isolated from mouse lung tissue using our previously published protocol with some modifications [12]. Using this protocol, we could show that EVs released in the lung during allergen-induced airway inflammation are enriched in inflammation-associated proteins, which are related with the activation of immune cells and an excessive immune response.

Allergen-induced inflammation, such as that which is seen in allergic asthma, is commonly associated with a T2 immune response. The process starts with allergen presentation, and this is followed by the release of T2 cytokines (e.g., IL-4, IL-5, IL-13) from Th2 cells and ILC2 that stimulate antibody production from B cells, activate mast cells, and attract immune cells such as eosinophils. We and others have previously shown that T2-induced (IL-4 and IL-13) inflammation increases the release of EVs from bronchial epithelial cells [9,22], and here, we could validate that EV release is also increased during T2-driven inflammation in vivo in mice (Figure 2C,D).

Our lung tissue-derived EVs contained the majority of the proteins previously identified in BALF-derived EVs [17] (Figure 3B), which demonstrates that our lung tissue-derived EVs represent previously isolated mouse airway-derived EVs well. Additionally, 3627 proteins not previously identified in the BALF-derived EVs were also quantified in our EVs. This may argue the benefit of using tissue samples when studying airway EVs because more proteins were identified. However, it is important to note that in the BALF-derived EV study, the mice were stimulated only once with poly (I:C), which is a model mimicking a viral infection and stimulates more of a T1 inflammation, which is different from our OVA protocol mimicking an eosinophilic airway inflammation stimulating a T2 inflammation and that runs over 19 days. It is possible that our OVA protocol stimulates a stronger response, resulting in more EVs and/or more or other proteins in the isolated EVs. Therefore, future studies will have to compare BALF-derived EVs and lung tissue-derived EVs from the same mouse models.

Importantly, the proteome of the EVs released in the lung during OVA stimulation was significantly altered and contained more immune-associated proteins compared to controls (Figure 3, Figure 4 and Figure 5). The most enriched protein in OVA-EVs was macrophage metalloelastase (also called MMP-12) (Figure 4F). MMP-12 is mainly expressed in alveolar macrophages, but it is also expressed in bronchial epithelial cells [23], and MMP-12 has been suggested to be pro-inflammatory in allergic inflammation and has been shown to be increased in allergic bronchial asthma in rats [24]. MMP-12 has also been suggested to be connected to the severity of asthma and chronic obstructive pulmonary disease (COPD) and to be involved in the tissue remodeling in these inflammatory respiratory diseases [25,26]. MMPs have been found in EVs before [27], but few studies have identified MMP-12 [27,28,29]. Furthermore, MMP-12 has been suggested to regulate the expression of NOS2 [30], which has been shown to be upregulated in the airway epithelium of patients with asthma and is the main producer of exhaled nitric oxide, which is a surrogate biomarker of eosinophilic airway inflammation [31,32]. Interestingly, NOS2 was upregulated 3-fold in the OVA-EVs compared to controls (Supplementary Table S1). We have previously shown that EVs isolated from the upper airways (nasal lavage fluid) contain functional NOS2 [33] and that the most upregulated protein in bronchial epithelial-derived EVs after T2 stimulation (IL-4 and IL-13) is NOS2 [9]. This shows that the OVA model is a good representation of the T2 inflammation that we detected in the bronchial epithelial cell cultures and shows that NOS2 is commonly found in airway-derived EVs both in vitro and in vivo in both mice and humans.

Two other proteins among the 15 most-enriched proteins were pendrin and arachidonate 15-lipoxygenase (also called ALOX15) (Figure 4F). Pendrin is an anion transporter and has been shown to be induced by the T2 cytokines IL-4 and IL-13 [34]. Furthermore, pendrin has been shown to be increased during airway hyper-responsiveness and to be associated with the mucus production in asthma and COPD [34,35], and we have previously shown that pendrin is enriched in both T2 and Th17-stimulated human bronchial epithelial cell-derived EVs [9]. ALOX15 has been shown to have increased expression in endobronchial biopsies from asthma patients compared to healthy controls, and one of the products of ALOX15, 15(S)-HETE, is increased in BALF from patients with severe asthma, and its levels are also associated with the number eosinophils present in the tissue [36]. Together, these findings suggest that airway-derived EVs contain proteins that can drive the pathology of asthma.

Several of the proteins enriched in OVA-EVs were associated with eosinophils. For example, C-C chemokine receptor type 3 (also called CCR3, Figure 4F) was increased. Eotaxin-1/CCL11, eotaxin-2/CCL24, and eotaxin-3/CCL26 (only in human) are ligands for the cell surface CCR3 and regulate eosinophil homing to tissues both in cooperation with IL-5 and through IL-5-independent pathways [37]. Eotaxins are increased in the airways of subjects with asthma [38], and we have previously shown that neutralizing antibodies against eotaxin-1/CCL11 and eotxin-2/CCL24 reduces the number of infiltrating eosinophils in the BALF of OVA-sensitized and challenged mice [13]. Interestingly, we also recently demonstrated that IL-33 driven airway eosinophilia was partly dependent on eotaxin-2/CCL24 levels. However, eotaxin-2/CCL24 levels were unaffected by IL-5 neutralization, suggesting that IL-33 driven eotaxin-2/CCL24 expression is independent of IL-5 [39]. Eosinophil peroxidase and eosinophil cationic protein 2 were also enriched in OVA-EVs (Figure 4F), and they are found within the eosinophil granulocytes, which are released upon eosinophil activation. These proteins are associated with host defense responses against helminth parasites, and they have also been suggested to have antibacterial activity [40]. However, when eosinophils are recruited to the lung during an allergen-induced airway inflammation, these proteins contribute to the inflammation, tissue damage, and remodeling observed in the asthmatic airway. This suggests that at least some of the EVs isolated from lung tissue in our allergic airway inflammation model are released by the infiltrating eosinophils observed in the lung (Figure 2A).

The proteins that were decreased in OVA-EVs compared to PBS-EVs were associated with mitochondria and metabolic processes. We currently do not know if this is due to a downregulation of these processes in the EV-producing cell or if it is due to these proteins not being loaded into the EVs to the same degree during eosinophilic airway inflammation. It has been shown that pollen extract induces mitochondrial dysfunction and that mitochondrial dysfunction increases the accumulation of eosinophils in the airway and increases the mucus production during allergic inflammation in a mouse model using ragweed pollen extract [41]. This suggests a connection between mitochondrial dysfunction and allergic diseases [42]. The downregulation of proteins associated with these basic biological processes of energy production and metabolism could also reflect a relative decrease in relation to the immune-associated proteins that are upregulated.

One of the proteins that was decreased in OVA-EVs was uteroglobin (also known as SCGB1A1 or CC10) (Figure 4C). Uteroglobin is a club cell-specific protein [43]. Club cells are non-ciliated cells in the small airways that serve as progenitors for ciliated cells, and they secrete proteins that protect the airway epithelium [44] and have anti-inflammatory functions [45,46]. The function of uteroglobin is not well understood, but it is decreased in BALF from asthma and COPD patients [45], and it has been suggested that airway remodeling leads to reduced club cell secretory function that increases the lung inflammation in chronic lung disease such as asthma and COPD [47]. Furthermore, uteroglobin downregulates the production of T2 cytokines such as IL-4, IL-5, and IL-13 in T cells and downregulates Th2 differentiation of T cells [48,49]. Additionally, we observed that that the proteins enriched in OVA-EVs were associated with immune cells (Figure 5A). Together, this suggests that few EVs are released by club cells during airway inflammation, and that most EVs are released by the immune cells that have migrated into the lung.

In conclusion, we show here that EVs can be isolated directly from lung tissue and that the EVs released during allergen-induced airway inflammation represent the inflammatory state under which they have been produced. This suggests that airway EVs promote the pathological processes associated with asthma, and these EVs might provide a better understanding of the disease-related mechanisms in asthma.

## 4. Materials and Methods

### 4.1. Animals

Male C57Bl/6 mice were purchased from Charles River (Sulzfeld, Germany) and kept under standard animal housing conditions with food and water ad libitum. Age and sex-matched mice at 6–10 weeks of age were used in all experiments. All the animal experiments were approved by the Gothenburg County Regional Ethical Committee, Gothenburg, Sweden (permit no. 126-2014 and 22-2016, approval date 24 June 2014).

### 4.2. Induction of Airway Inflammation

We followed our previously established protocol to induce acute allergic airway inflammation with OVA with minor changes [15]. Briefly, mice were sensitized to 8 μg chicken ovalbumin (OVA; Sigma-Aldrich, St. Louis, MO, USA) bound to 4 mg aluminium hydroxide (Sigma-Aldrich) in phosphate-buffered saline intraperitoneally (i.p.) at day 1 and 6. On days 14–18, the mice were challenged once a day intranasally (i.n.) with 100 μg OVA in 25 μL PBS to induce allergic airway inflammation (12 OVA/OVA mice). The control group was exposed to PBS only during these 5 days (10 OVA/PBS mice).

### 4.3. Sample Collection

The animals were anesthetized 24 h after the last OVA challenge using a mixture of xylazin (130 mg/kg; Rompun, Bayer, Germany) and ketamine (670 mg/kg; Ketalar, Apoteket AB, Motala, Sweden). The mice were sacrificed by puncturing the right heart ventricle and removal of the blood. Then, the mice were tracheotomized, and BALF was harvested by instilling 0.25 mL and 0.20 mL of PBS through the tracheal cannula, followed by gentle aspiration. The BALF was later used for differential cell count analysis to confirm the airway inflammation due to the allergen challenge. Next, all five lobes, without any connective tissue, were removed and stored on ice in Hanks balanced salt solution (Sigma-Aldrich) and directly transferred for EV isolation.

### 4.4. Differential Cell Count

The BALF was centrifuged at 170× *g* for 10 min, and the cells were resuspended in PBS and counted. Approximately 10,000–50,000 cells were used per slide, and the samples were centrifuged 425× *g*, for 6 min (Shandon Cytospin 3 centrifuge) and stained with May–Grünwald–Giemsa (Histolab, Askim, Sweden) according to the manufacturer’s protocol. Eosinophils were assessed by histological examination and counted.

### 4.5. EV Isolation

EVs were isolated from the lung tissue according to our previously published protocol with some minor changes [12]. Briefly, the tissues were weighed, and RPMI-1640 medium (Sigma Aldrich) was added to each lung in the Petri dish, and the tissues were sliced into small pieces (approximately 2 mm × 2 mm). Then, the small pieces were incubated for 30 min at 37 °C in RPMI-1640 medium supplemented with collagenase D (2 mg/mL, Roche, Basel, Switzerland) and DNase I (40 U/mL, Roche). Next, the EV-containing media was filtered through a 70 µm cell strainer (Fisher Scientific, Gothenburg, Sweden) to remove tissue pieces. To further remove cells and debris, the samples were centrifuged at 500× *g* for 10 min and then at 2000× *g* for 20 min twice to remove apoptotic bodies and other large EVs. The supernatant was ultracentrifuged at 52,000 rpm (TLA100.3 rotor, Beckman Coulter, Brea, CA, USA) for 60 min to isolate the EVs. Isolated EVs were re-suspended in PBS, and the samples were pooled two and two (final volume 300 µL) so that the 12 OVA/OVA samples ended up as six samples and the 10 OVA/PBS samples ended up as five samples. The samples were mixed with 3.7 mL 50% iodixanol (Optiprep, Sigma Aldrich) to reach a final volume of 4 mL, and 4 mL of 30% and 4 mL of 10% iodixanol were then layered on top. The density cushions were ultracentrifuged at 28,000 rpm for 2 h (SW 41 rotor, Beckman Coulter). Then, the EVs were collected in the interphase between the 10% and 30% iodixanol layers.

### 4.6. Transmission Electron Microscopy

Formvar/carbon-coated nickel grids (Ted Pella, Inc., Redding, CA, USA) were glow discharged prior to incubation with the samples for 15 min. Then, samples were sequentially fixed in 2% paraformaldehyde and 2.5% glutaraldehyde prior to being negative-stained with 2% uranyl acetate. The grids were examined using a Tecnai T12 transmission electron microscope with a Ceta CMOS 16M camera (FEI, Hillsboro, OR, USA).

### 4.7. Nanoparticle Tracking Analysis

The particle concentration of the EVs was measured using a ZetaView^®^ PMX 120 device (Particle Metrix, Meerbusch, Germany). EV preparations were thawed immediately before measurements and were diluted in 2 mL of 0.2 µm-filtered PBS (2000-fold dilution) before being injected into the instrument. The measurement was carried out three times at all 11 different positions, the video quality was set to medium, and the camera sensitivity was set to 80. Data were analyzed using the ZetaView^®^ analysis software with a minimum size of 10, a maximum size of 1000, and a minimum brightness of 30.

### 4.8. Protein Measurement

The protein concentration was determined using a BCA assay kit according to the manufacturer’s instructions (Pierce BCA™ Protein Assay Kit (Thermo Fisher Scientific, Waltham, MA, USA).

### 4.9. Western Blot

Samples (2 µg of protein) were prepared in Laemmli sample buffer (Bio-Rad Laboratories, Hercules, CA, USA) with 2-Mercapthoethanol (Sigma-Aldrich) Sample Reducing Agent. Samples were heated to 95 °C for 5 min and loaded onto a 4–20% polyacrylamide Mini-PROTEAN TGX gel (Bio-Rad Laboratories, Hercules, CA). After transferring to a PVDF membrane (Bio-Rad Laboratories), the membrane was blocked with EveryBlot Blocking buffer (Bio-Rad Laboratories) for 10 min at RT and then incubated with the primary antibody (anti-flotillin-1, 1:1000 dilution, clone EPR6041, Abcam, Cambridge, UK) diluted in EveryBlot Blocking buffer at 4 °C overnight. Then, the membrane was washed three times with 0.1% TBS-Tween and was further incubated with the secondary antibody (donkey anti-rabbit IgG HRP-linked F(ab)2 fragment, 1:5000 dilution; catalogue no. NA9340V, GE Healthcare, Buckinghamshire, UK) for 1 h at RT. Then, the membrane was washed four times for 5 min in 0.1% TBS-Tween and analyzed with the SuperSignal West Femto maximum sensitivity substrate (Thermo Fisher Scientific) on a ChemiDoc Imaging System (Bio-Rad Laboratories).

### 4.10. Sample Preparation and Digestion for LC-MS/MS

The proteomic analysis was performed at The Proteomics Core Facility at Sahlgrenska Academy, Gothenburg University. The samples were digested with trypsin using the filter-aided sample preparation method [50]. Briefly, samples were reduced with 100 mM dithiothreitol at 60 °C for 30 min, transferred to 30 kDa MWCO Pall Nanosep centrifugation filters (Sigma-Aldrich), washed several times with 8 M urea, and washed once with digestion buffer prior to alkylation with 10 mM methyl methanethiosulfonate in digestion buffer for 30 min. Digestion was performed in 50 mM TEAB and 1% sodium deoxycholate (SDC) buffer at 37 °C by the addition of 0.3 µg Pierce MS grade trypsin (Thermo Fisher Scientific) and incubated overnight. An additional portion of trypsin was added and incubated for another 2 h, and peptides were collected by centrifugation. Digested peptides were labeled using tandem mass tag (TMT) 11-plex isobaric mass tagging reagents (Thermo Fisher Scientific) according to the manufacturer’s instructions. Sodium deoxycholate was removed by acidification with 10% trifluoroacetic acid, and the sample was further purified using High Protein and Peptide Recovery Detergent Removal Resin (Thermo Fisher Scientific) according to the manufacturer’s instructions. The combined purified samples were pre-fractionated into 40 fractions with basic reversed-phase chromatography using a Dionex Ultimate 3000 UPLC system (Thermo Fisher Scientific). Peptide separations were performed using a reversed-phase XBridge BEH C18 column (3.5 μm, 3.0 × 150 mm, Waters Corporation) and a linear gradient from 3% to 40% solvent B over 18 min followed by an increase to 100% B over 5 min and 100% B for 5 min at a flow rate of 400 µL/min. Solvent A was 10 mM ammonium formate buffer at pH 10.00, and solvent B was 90% acetonitrile and 10% 10 mM ammonium formate at pH 10.00. The fractions were concatenated into 20 fractions, dried, and reconstituted in 3% acetonitrile and 0.2% formic acid.

### 4.11. NanoLC-MS/MS Analysis and Database Search

Each fraction was analyzed on an Orbitrap Fusion Tribrid mass spectrometer (Thermo Fisher Scientific) interfaced with an nLC 1200 liquid chromatography system. Peptides were trapped on an Acclaim Pepmap 100 C18 trap column (100 μm × 2 cm, particle size 5 μm, Thermo Fischer Scientific) and separated on an in-house constructed analytical column (330 mm × 0.075 mm I.D.) packed with 3 μm Reprosil-Pur C18-AQ particles (Dr. Maisch, Germany) using a linear gradient from 5% to 35% B over 75 min followed by an increase to 100% B for 5 min and 100% B for 10 min at a flow rate of 300 nL/min. Solvent A was 0.2% formic acid in water and solvent B was 80% acetonitrile in 0.2% formic acid. Precursor ion mass spectra were acquired at 120,000 resolution, and MS/MS analysis was performed in a data-dependent multinotch mode where the CID spectra of the most intense precursor ions were recorded in the ion trap at a collision energy setting of 35 for 3 s (the “top speed” setting). Precursors were isolated in the quadrupole with a 0.7 *m*/*z* isolation window, charge states 2 to 7 were selected for fragmentation, and dynamic exclusion was set to 60 s and 10 ppm. MS3 spectra for reporter ion quantitation were recorded at 50,000 resolution with HCD fragmentation at a collision energy of 65 using the synchronous precursor selection.

The data files for each set were merged for identification and relative quantification using Proteome Discoverer version 1.4 (Thermo Fisher Scientific). The search was against the Mus musculus SwissProt Database version November 2017 (Swiss Institute of Bioinformatics, Lausanne, Switzerland) using Mascot 2.5 (Matrix Science) as the search engine with a precursor mass tolerance of 5 ppm and a fragment mass tolerance of 0.6 Da. Tryptic peptides were accepted with zero missed cleavages, variable modifications of methionine oxidation and fixed cysteine alkylation, and TMT-labeled modifications of N-terminal and lysine were selected. A percolator was used for PSM validation with the strict FDR threshold of 1%, and the quantified proteins were filtered at 5% FDR and grouped by sharing the same sequences in order to minimize redundancy. TMT reporter ions were identified in the MS3 HCD spectra with 3 mmu mass tolerance, and the TMT reporter intensity values for each sample were normalized to the total peptide amount in Proteome Discoverer 1.4. Only peptides unique for a given protein were considered for the relative quantification. The sum of the control samples was used as the denominator and for calculation of the ratios.

### 4.12. Bioinformatics and Statistical Analysis

Where appropriate, data are expressed as the mean, and individual values are shown. Statistical analysis was performed with Student’s non-paired *t*-test. Qlucore Omics Explorer (Qlucore, Lund, Sweden) was used for the principal component analysis, multi-group comparison, and unsupervised hierarchical clustering. DAVID was used to determine the associated cellular components and biological processes (https://david.ncifcrf.gov/, access date: 19 September 2018) [51,52]. The mouse protein accession IDs were converted to human proteins with bioDBnet (https://biodbnet-abcc.ncifcrf.gov/db/dbOrtho.php, access date; 18 February 2021) so that FunRich (http://www.funrich.org/, access date; 18 February 2021) could be used to analyze the sites of expression of the proteins.

## Figures and Tables

**Table 1:** Common EV proteins.

Protein Group	Proteins
Rabs	Rab-1A, -1B, -2A, -3A, -3D, -4B, -5A, -5B, -5C, -6A, -6B, -7A, -8A, -8B, -9A, -10, -11B, -12, -13, -14, -18, -21, -23, -24, -27A, -27B, -31, -32, -33B, -34, -35, -38, -43
Annexins	Annexin A1, A2, A3, A4, A5, A6, A7, A8, A11
Tetraspanins	CD9, CD37, CD53, CD63, CD81, CD82, CD151, TSPAN6, TSPAN7, TSPAN8, TSPAN9, TSAPN14, TSPAN15, TSPAN18, TSPAN31
Common EV markers	ADAM10, Ezrin, Cofilin-1, Flotillin-1, Flotillin-2, Profilin-1, Profilin-2, 14-3-3 (beta/alpha, zeta/delta, epsilon, eta, gamma, theta, sigma)
ESCRT	ESCRT-0: STAM2 ESCRT-I: TSG101, VPS37C, MVB12A ESCRT-II: VPS25, VPS36 ESCRT-III: CHMP2A, CHMP2B, CHMP6, CHMP3, CHMP4B CHMP4C, CHMP5, CHMP1A, CHMP1B, IST1 ESCRT accessory: VSP4A, VSP4B, ALIX

## Data Availability

The mass spectrometry proteomics data will be deposited to the ProteomeXchange Consortium via the PRIDE [53] partner repository with the dataset identifier PXD025688.

## Supplementary Materials

The following are available online at https://www.mdpi.com/article/10.3390/ijms22094718/s1, Table S1: List of all quantified proteins, Table S2: Proteins from the multi-group comparison in Figure 3D.
